# Transfer of the assassin bug *Helonotus pallidulus* Walker to the genus *Heza* Amyot & Serville (Hemiptera, Heteroptera, Reduviidae, Harpactorinae, Harpactorini)

**DOI:** 10.3897/zookeys.872.35137

**Published:** 2019-08-26

**Authors:** Hélcio R. Gil-Santana, Michael D. Webb

**Affiliations:** 1 Laboratório de Diptera, Instituto Oswaldo Cruz, Av. Brasil, 4365, 21040-360, Rio de Janeiro, Brazil Instituto Oswaldo Cruz Rio de Janeiro Brazil; 2 Department of Life Sciences (Insects), The Natural History Museum, Cromwell Road, London SW7 5 BD, UK The Natural History Museum London United Kingdom

**Keywords:** Ecuador, Neotropical region, new combination, *Ploeogaster pallidulus*

## Abstract

Based on examination of its holotype, *Helonotus pallidulus* Walker, 1873 is transferred to the genus *Heza* Amyot & Serville, 1843 with the resulting new combination: *Heza pallidula* (Walker, 1873), **comb. nov.** (Hemiptera, Heteroptera, Reduviidae, Harpactorinae, Harpactorini). A comparison with the similar *Heza ventralis* Stål,1872 is provided.

## Introduction

Harpactorinae is the largest subfamily of Reduviidae and is represented by the tribes Apiomerini and Harpactorini in the Neotropical region (Gil-Santana et al. 2015). Harpactorini is the most diverse Reduviidae tribe with 53 recognized genera in the Neotropical region (Forero et al. 2008, McPherson and Ahmad 2011, Forero 2011, 2012, Swanson 2012, Gil-Santana 2015, Gil-Santana et al. 2015, Zhang et al. 2016, Lapischies et al. 2019). The only key to separate the genera is by Stål (1872), now badly out of date (Forero 2011). Of all genera of Harpactorini in the Neotropical region, just a few have been revised taxonomically or redescribed.

Walker (1873) described *Helonotus pallidulus* Walker, 1873 based on a specimen (described as a male) from “Cuenca”, thereby placing a New World species in the Old World genus, *Helonotus* Amyot & Serville, 1843. In the catalog of Lethierry and Severin (1896) this species was included among the Harpactoridae “Genera et species Harpactoridarum subfam. incerti loci systematici”. Distant (1903) included it in the New World genus *Ploeogaster* Amyot & Serville, 1843, stating that he had “not sufficiently compared this [*Ploeogaster pallidulus*] with other species of the genus [*Ploeogaster*] to say that it is not a synonym.” Since then, *Ploeogaster pallidulus* has only been cited in catalogues (Wygodzinsky 1949, Maldonado Capriles 1990).

In the current work, we confirm that the holotype of *Helonotus pallidulus*, deposited in the Natural History Museum, London, does not belong to either *Helonotus* or *Ploeogaster*. The main differences being that in *P. pallidulus* a postantennal spine is present (rather than a short tubercle in *Ploeogaster* or both absent in *Helonotus*) and the disc of the hind lobe of the pronotum has a pair of spines in *P. pallidulus* (rather than a pair of tubercles in *Ploeogaster* and one or two pairs of tubercles in *Helonotus*) (Amyot and Serville 1843, Malipatil 1986, 1991). However, as commented in detail below, the above characteristics observed in the holotype of *H. pallidulus* are entirely concordant with members of the New World genus *Heza* Amyot & Serville, 1843, a genus revised by Maldonado Capriles (1976, 1983).

## Material and methods

**Figures 1–4.**
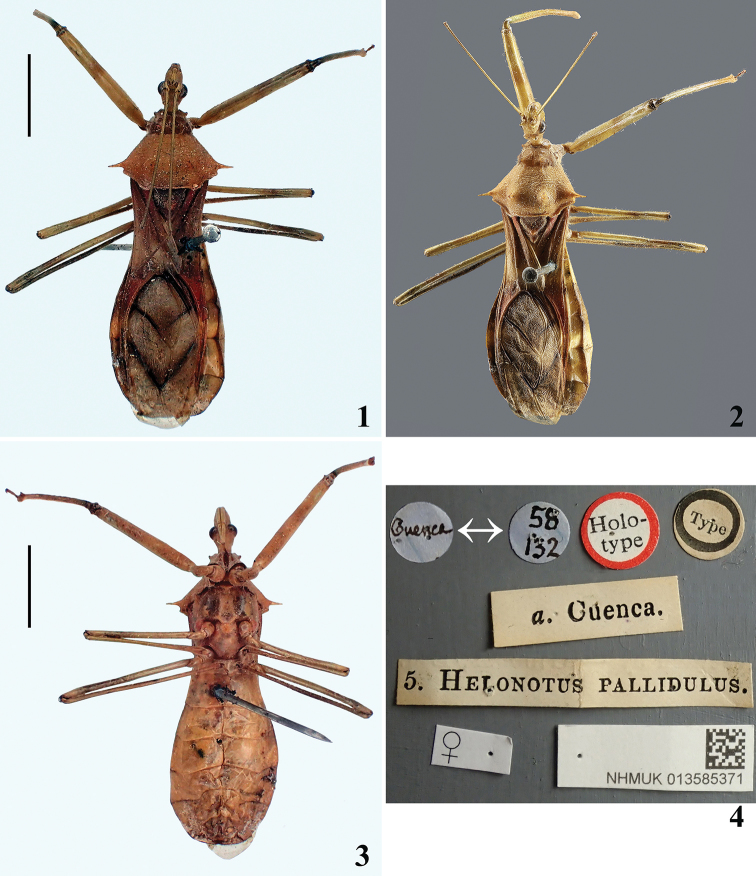
*Heza pallidula* (Walker, 1873), comb. nov., female holotype. **1** Dorsal view **2** dorsal view (with antennae and left fore leg repositioned) **3** ventral view **4** labels. Scale bars: 5.0 mm (**1, 3**).

**Figures 5, 6.**
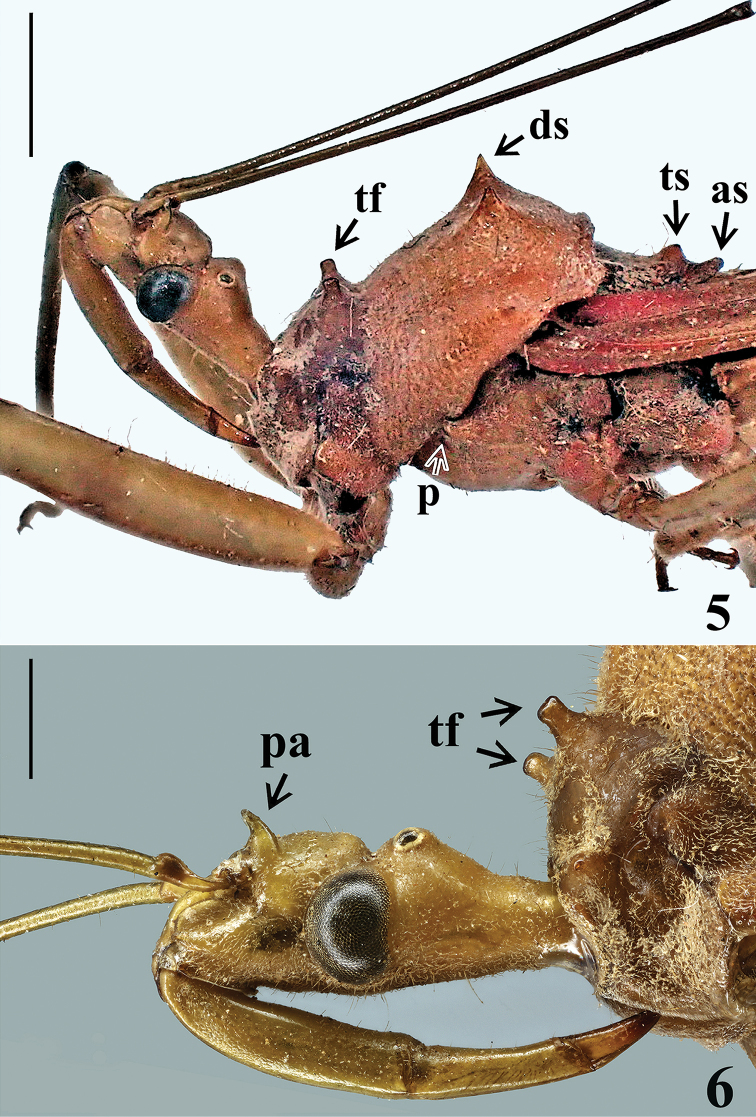
Head and thorax of *Heza pallidula* (Walker, 1873), comb. nov., female holotype, lateral view. **6** Antennae repositioned (**as**, apex of scutellum, **ds**, discal spine of hind lobe, **p**, plica, **pa**, postantennal spine, **tf**, discal tubercle of fore lobe, **ts**, tubercle of disc of scutellum). Scale bars: 2.0 mm (**5**); 1.0 mm (**6**).

For the present study, the holotype of *Helonotus pallidulus*, deposited in the Natural History Museum, London, United Kingdom (BMNH), was directly examined (Figs 1–6).

After imaging the specimen (Figs 1, 3–5), the antennae were relaxed and moved forward in order to obtain a clearer image of the postantennal spine. Additional photographs were taken for a better visualization of the pronotum and postantennal spines (Figs 2, 6).

General morphological terminology mainly follows Schuh and Slater (1995). However, the [visible] segments of the labium are numbered as II to IV, given that the first segment is lost or fused to the head capsule in Reduviidae (Weirauch 2008, Schuh et al. 2009).

When describing label data, a slash (/) separates the lines and a double slash (//) different labels.

## Results

### Taxonomy

#### Subfamily Harpactorinae

##### Tribe Harpactorini

###### Heza pallidula

Taxon classificationAnimaliaHemipteraReduviidae

(Walker, 1873) comb. nov.

32D3A8140778544FB4194D2F6A2A37EE

Figs 1–4 , 5–6

Helonotus pallidulus Walker, 1873: 90 [description]; Lethierry and Severin 1896: 200 [catalog, among “Genera et species Harpactoridarum subfam. incerti loci systematic”].
Ploeogaster pallidulus ; Distant 1903: 251 [checklist]; Wygodzinsky 1949: 44 [catalog]; Maldonado Capriles 1990: 259 [catalog].

####### Notes.

This species was described from a single specimen (holotype) for which Walker (1873) provided the following data: “*a.* Cuenca. From Mr. Fraser’s collection”. The reference to 58/132 on the label noted above (Fig. 4) refers to an entry in the BMNH register for 1858 132: Cuenca (Province of Ecuador) collected by Fraser. The type specimen, originally recorded as male is in fact female (Fig. 3).

####### Material examined.

*Helonotus pallidulus*, female **holotype**, [Ecuador]: 5. Helonotus pallidulus. // *a.* Cuenca. // Cuenca [opposite side of same label]: 58/132// Holo- / type [rounded label with red circle] // ♀ // Type [rounded label with blackish circle] // QR CODE / NHMUK 013585371 (BMNH).

####### Redescription.

Female (Figs 1–6). Measurements: total length: 22.0 mm; maximum width of abdomen: approximately 7.5 mm.

####### Coloration.

General color pale brownish to brownish red (Figs 1, 2); pale yellowish on ventral surface of thorax and abdomen (Fig. 3). Antenna with distal fourth of segments I and II darkened (other segments absent). Legs somewhat lighter, with fuscous ill-defined submedian marking on fore femur and submedian and distal markings on remaining femora; apices of femora somewhat darkened. Scutellum darkened, with apex reddish brown. *Hemelytra*: corium with base, apical portion at level of membrane and basal half of clavus reddish, median portion somewhat paler, grayish; membrane pale grayish, approximately basal half of anal and cubital veins and median vein mostly blackish (Fig. 1). Meso and metapleura reddish brown (Fig. 5). Meso and metasterna generally paler; stridulitrum and lateral portions of mesosternum darkened (Fig. 3). *Abdomen*: connexiva faintly darkened dorsally at basal half of segments IV–VII (Fig. 1); sternites generally yellowish to pale orange (Fig. 3).

####### Structure.

*Head* (Figs 5, 6): elongated with a well marked transverse sulcus, shorter than pronotum, and a pair of spines just behind antennal bases; these spines (postantennal spines) are conspicuously bowed with the apex directed slightly anteriad (Fig. 6, pa). Eyes globose, glabrous, projecting laterally, rounded in dorsal view and suboval in lateral view. Ocelli elevated, closer to eyes than to each other. Antennal segments I–II (other absent) straight, slender, segment I somewhat longer than head and pronotum combined; segment II quite shorter than head. Labium stout, moderately curved, segment II (first visible) thickest and longest; segment IV, approximately half as long as segment III, tapering (Fig. 6). *Thorax*. Pronotum: hind lobe approximately two and half times longer than fore lobe, with its maximum width (at posterior margin) somewhat more than twice that of fore lobe; anterior collar inconspicuous; anterolateral angles pronounced; transverse sulcus well marked (Figs 1, 2). Fore lobe divided in two sublobes by shallow median longitudinal depression, with a blunt tubercle on disc of each sublobe (Figs 1–2, 5–6, tf). Hind lobe finely transversely corrugate, with four sharp spines (Figs 1, 2); of these, the discal spines (Figs 2, 5, ds) shorter, directed dorsad, larger at base, preceded by an inconspicuous carina; lateral spines relatively long, conspicuous, horizontally directed laterally (Figs 1, 2). Posterior margin curved laterally, with a pair of rounded prominences at level of base of clavus; margin between these prominences slightly curved (Figs 1, 2). Scutellum with a rounded median tubercle on disc (Fig. 5, ts) just before apex, which is rounded and somewhat obliquely elevated (Fig. 5, as). Mesopleura with a well-developed plica (Fig. 5, p), i.e., a small raised tubercle over posterior margin of propleuron. Prosternum almost entirely anterior to fore coxa and shorter than them, with its median portion occupied by stridulitrum. Mesosternum flattened, somewhat depressed at median portion, larger than metasternum. Legs: fore coxae contiguous to each other; middle and hind coxae distant from each other by a distance approximately equivalent to twice width of each coxa (Fig. 3). Apices of all femora with a pair of lateral small tubercles. Femora generally straight; fore femora thickened (Figs 1–3), approximately thrice thicker than middle and hind femora; middle and hind femora slender, slightly dilated subapically; hind femora longest, middle femora shortest. Fore tibiae curved inwards in distal half, somewhat enlarged at apex, with a dorsal spur apically; middle and hind tibiae generally straight, slender. Hemelytra surpassing apex of abdomen for a short distance (Figs 1–3). *Abdomen* spatulate, gradually widening to apex of segment V and then slightly narrowing to form a roughly truncate apex at last segment (Fig. 3). All connexival segments without spines.

####### Vestiture.

Integument generally covered by short thin adpressed setae, more numerous on thorax (Fig. 2). Legs generally covered with longer straight erect setae; fore legs with trochanter, femur and tibia ventrally with a dense pubescence formed by short erect thin setae. Membrane of hemelytra glabrous.

## Discussion

The previous placement of this species in *Ploeogaster* by Distant (1903) is incorrect, as some characteristics of the latter are different, such as the absence of postantennal spines (only short postantennal tubercles are present) and the hind lobe of the pronotum without four sharp spines; only with two (lateral) spines, while on the disc, instead of spines, there is a pair of rounded tubercles. Had Distant (1903) “sufficiently compared [*Helonotus pallidulus*] with other described species”, he possibly would have placed the species in a different genus.

The transfer of *Helonotus pallidulus* to *Heza* is in accordance with the following features considered as diagnostic for the genus (Amyot and Serville 1843, Maldonado Capriles 1976): head shorter than pronotum, with postantennal spines (Figs 1, 2, 5, 6); pronotum: fore lobe with a pair of blunt or pointed spines on the disc (Figs 5, 6, tf), hind lobe with four sharp spines (Figs 1, 2, 5, ds); mesopleura with a plica (Fig. 5, p); abdomen spatulate, somewhat widened before apex but behind middle of abdomen (Fig. 3).

Besides confirming that the characteristics of *Heza pallidula* are entirely in accordance with those attributed to *Heza*, it is also possible to conclude by consulting the revision, keys, descriptions and redescriptions provided by Maldonado Capriles (1976, 1983) and Maldonado Capriles and Brailovsky (1983) that this species does not correspond to any previously described species of this genus and must be maintained as a valid species.

*Heza pallidula* seems to be most similar to *Heza ventralis* Stål, 1872. The latter was described by Stål (1872) and a photo of its type has been made freely available by the Swedish Museum of Natural History (Naturhistoriska riksmuseet, NRM) at http://www2.nrm.se/en/het_nrm/v/heza_ventralis.html. Maldonado Capriles (1976) redescribed *H. ventralis* based only on female specimens. The females of *H. pallidula* and *H. ventralis* share some structural similarity, such as approximate length and shape of the body, similar size of postantennal spines (Fig. 6), and both size and shape of the pronotal spines (Figs 1–3, 5, 6), and the absence of spines on all connexival segments; the three latter characteristics shared with very few species of the genus. Most species of *Heza* have one or more connexival segments spined on its apical angle (Maldonado Capriles 1976, 1983, Maldonado Capriles and Brailovsky 1983). On the other hand, the sericeous white areas on the thorax and more extensively on abdominal sternites are a clear-cut characteristic to separate *H. ventralis* from all other species in the genus, including *H. pallidula*. In *H. pallidula*, the reddish portions of the hemelytra (base and distal portion of corium and basal half of the clavus) and connexivum faintly darkened dorsally at basal half of segments IV–VII (Fig. 1) differ from the uniformly brownish-gray clavus, corium and connexivum of *H. ventralis*. Whereas the postantennal spine is conspicuously bowed with the apex slightly directed anteriad in *H. pallidula* (Fig. 6), it is straight and directed dorsad in *H. ventralis* (Stål 1872, Maldonado Capriles 1976).

The original spelling of the specific name *pallidulus* was changed to *pallidula* in the new combination because, according with the International Code of Zoological Nomenclature (ICZN 1999), if a species-group name is a Latin adjective in nominative singular, it “must agree in gender with the generic name with which it is at any time combined” (Art. 31.2).

## Supplementary Material

XML Treatment for Heza pallidula
